# An Explorative Study to Assess the Suicidal Risk Amongst Infertile Patients

**DOI:** 10.1192/j.eurpsy.2023.2355

**Published:** 2023-07-19

**Authors:** B. Ghosh Dastidar

**Affiliations:** Psychiatry, Imperial College NHS Trust CNWL, London, United Kingdom

## Abstract

**Introduction:**

This given study was designed to qualitatively and quantitatively examine the Psychosocial – emotional consequences of infertility on female infertile patients . Suicidal risk amongst infertile patients was a incidental yet significant finding with 25 percent of the study population reporting a positive result by the MINI scale. There are very few studies conducted in the Indian context that analyses the psychosocial aspects of infertility and the impact of ART treatment on the quality of life.

The finding in our study indicates that both infertility and stress associated with ART treatment contributes to psychological turmoil namely depression , anxiety, psychopathology and quality of life impairment in addition to suicidal ideation and suicidal risk.

**Objectives:**

Aims

To assess the psychosocial impact of infertility amongst female infertile patients including suicidal risk/ suicidal ideation in the given study population.

**Methods:**

A total of 300 women attending the Obstetrics and Gynecology out patients department of a tertiary hospital in Kolkata, India were selected by simple random sampling. 100 fertile women attending the routine ante natal clinic were selected as cases and 100 infertile women seeking fertility treatment were selected as controls. 100 women didn’t follow up with the study. The following questionnaires were administered to both case and control group- BAI, BDI, SCL-90-R, SF-36, MINI and socio demographic proforma ; by trained clinic psychologist .

The raw scores & adjusted scores were analysed statistically by SPSS using the following tests, independent t test, chi square test and Z test.

**Results:**

The results of the MINI scale indicate that up to 25% of the infertile cohort suffer from suicidal risk/ suicidal ideation which is statistically significant in comparison to the control group.

The other psychosocial parameters are also statistically significant in the case in comparison to the control population.

**Conclusions:**

Although the psychosocial impact of infertility has been well researched and documented. Few studies have been conducted globally which assess suicidal risk amongst infertile patients.

Our results corroborate earlier studies such as the Danish administrative population-based registry study by Trille Kristina Kjaer et al which found a causative link between infertility and suicidal risk.

Further research is needed in this direction

**Disclosure of Interest:**

None Declared

